# Supplementary material for: Gut Microbiome in Children from Indigenous and Urban Communities in México: Different Subsistence Models, Different Microbiomes
Source: Microorganisms. 2020 Oct 16;8(10):1592. doi: 10.3390/microorganisms8101592 (PMC7602701; doi:10.3390/microorganisms8101592)
Supplement: Supplementary file 1 [file microorganisms-08-01592-s001.pdf]

Supplementary Materials:

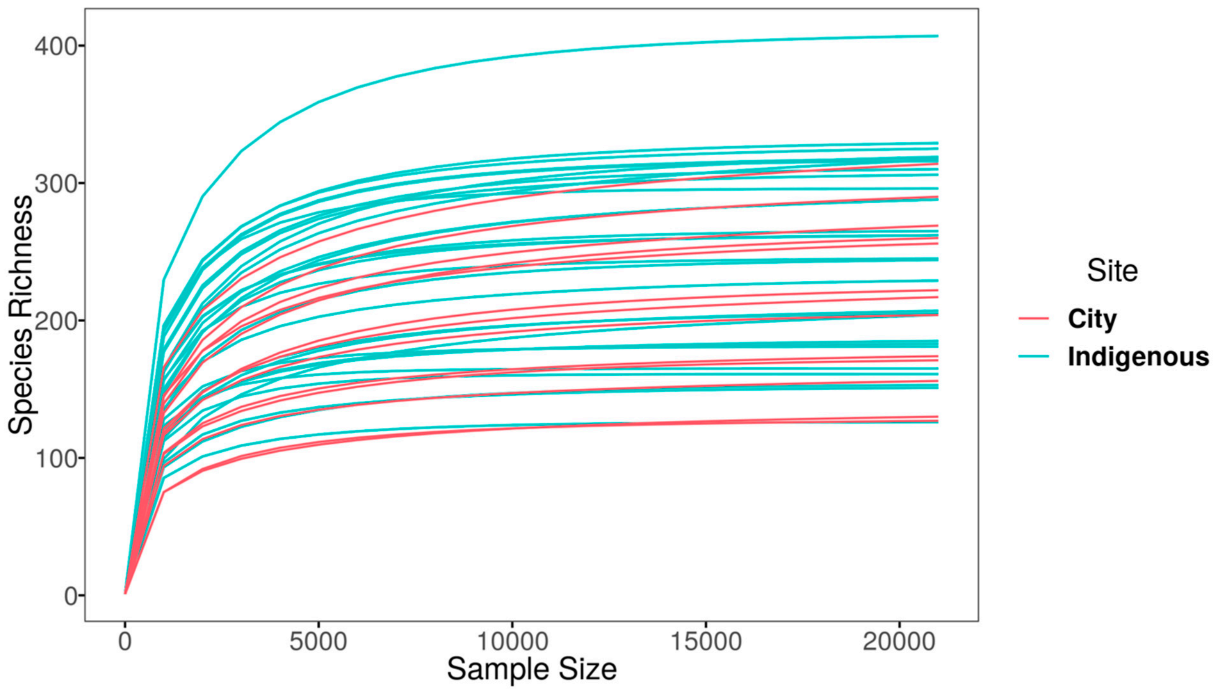

Figure S1. Accumulation curve of 16S amplicon libraries from each individual in the study. Mexico City and Me'phaa children are represented in pink and blue respectively. Phylotypes were based on ASV sequence identity.

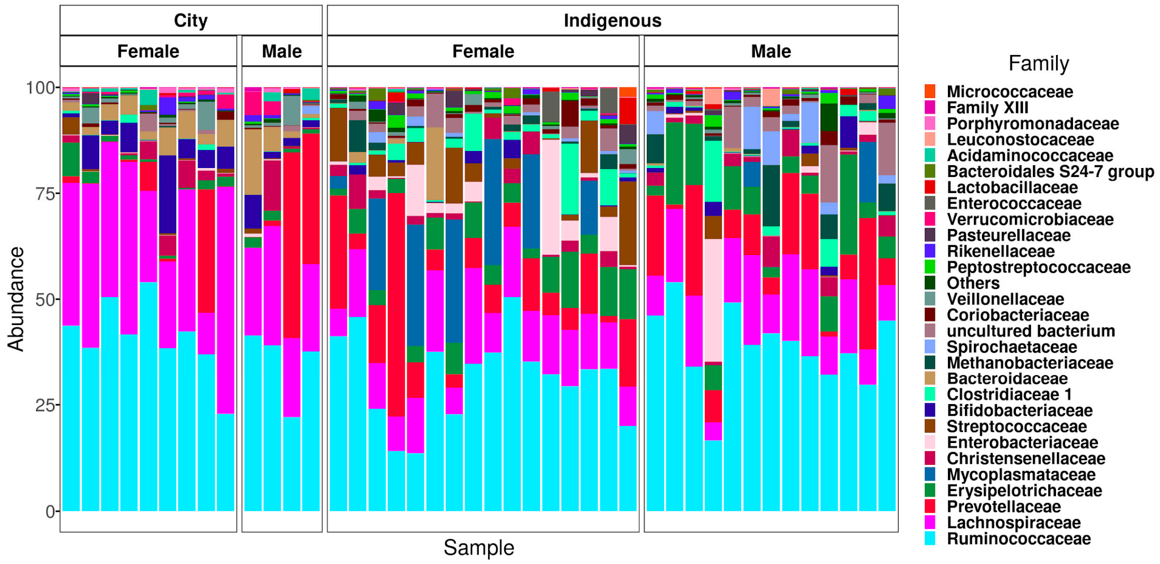

Figure S2. Distribution of bacterial composition (16S rDNA V4) at family level of male and female children from Mexico City and the Me'phaa community, families with relative abundances < 1 % were agglomerated in the "Others" category.

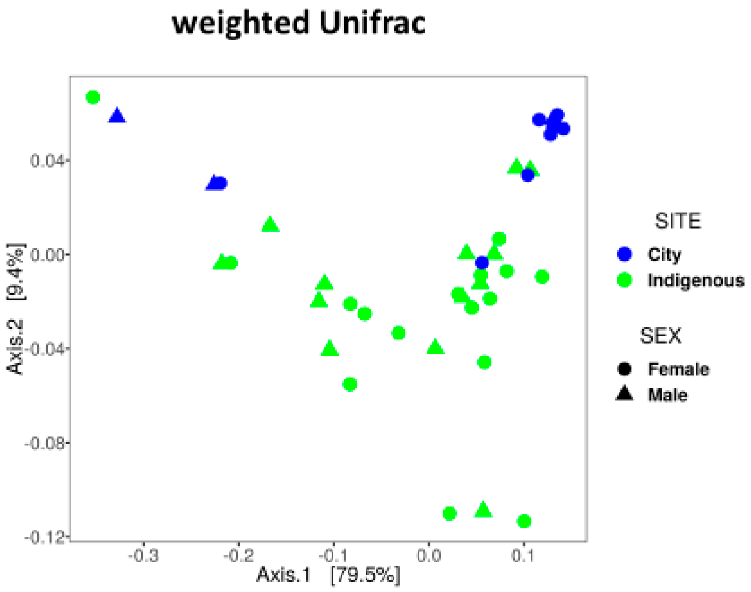

Figure S3. Weighted Unifrac Analysis. The GM of two communities represented in this study (City = Mexico City (blue); indigenous = Me´phaa (green)). Sex is showed with a circle for males and a triangle for females (PERMANOVA: F=2.79; p= 0.08).

Mexico City	
Phylum	Relative abundance
Firmicutes	77.103613522
Bacteroidetes	14.8497693691
Actinobacteria	5.3471919457
Verrumicrobia	1.1493073747
Proteobacteria	0.744833246
Tenericutes	0.5744705016
Spirochaetae	0.1458158544
Cyanobacteria	0.0560548385
Euryarchaeota	0.027477862
Fusobacteria	0.000732743
Elusimicrobia	0.0003663715
Saccharibacteria	0.0003663715
Indigenous (Me'phaa)	

Phylum	Relative abundance
Firmicutes	68.0491496599
Bacteroidetes	12.0734693878
Tenericutes	8.7302721088
Proteobacteria	4.4933673469
Actinobacteria	2.4447278912
Euryarchaeota	2.1181972789
Spirochaetae	1.6964285714
Cyanobacteria	0.2472789116
Verrumicrobia	0.0826530612
Chloroflexi	0.0163265306
Elusimicrobia	0.0120748299
Deinococcus-Thermus	0.0079931973
Acidobacteria	0.0071428571
Fibrobacteres	0.0049319728
Planctomycetes	0.0040816327
Gemmatimonadetes	0.0035714286
Fusobacteria	0.0034013605
Latescibacteria	0.0022108844
Nitrospirae	0.0011904762
Lentisphaerae	0.0010204082
Hydrogenedentes	0.0003401361
Aminicenantes	0.000170068

Supplementary Table S1. Relative abundances of the most abundant phyla found in fecal samples from children in Mexico City and the Me'phaa communities.

Individual Code	Group	Sex	Age	Height (m)	Weight (kg)	BMI
A27	Mexico City	M	6	1.26	22.7	14.29

A36	Mexico City	M	9	1.34	28.2	15.7
A09	Mexico City	F	7	1.21	27.4	18.71
A07	Mexico City	F	6	1.22	23.9	16.05
A35	Mexico City	M	11	1.43	37	18.09
A13	Mexico City	F	9	1.37	42.6	22.69 *
A31	Mexico City	F	5	1.14	17.4	13.38
A20	Mexico City	F	10	1.44	42.6	20.54
A26	Mexico City	F	9	1.46	37	17.35
A16	Mexico City	F	8	1.34	25.9	14.42
A05	Mexico City	F	6	1.16	22.9	17.01
A29	Mexico City	F	9	1.39	42.6	22.04 *
A24	Mexico City	M	6	1.16	21	15.6
103	Me'phaa	F	7	1.07	17.6	15.37
82	Me'phaa	F	5	1.02	15.4	14.8
101	Me'phaa	M	9	1.2	21.5	14.93
136	Me'phaa	F	8	1.19	20.5	14.4
151	Me'phaa	F	6	0.9	14.7	18.14
96	Me'phaa	M	8	1.19	19.7	13.9
114	Me'phaa	M	6	1.04	18.1	16.73
76	Me'phaa	M	6	1.18	20.7	14.86
135	Me'phaa	M	10	1.26	23	14.48
92	Me'phaa	F	6	1.09	16.2	13.63
117	Me'phaa	F	9	1.26	26.4	16.62
131	Me'phaa	M	9	1.27	27.7	17.17
109	Me'phaa	F	9	1.22	21.2	14.24
102	Me'phaa	M	8	1.12	17.8	14.19
95	Me'phaa	M	10	1.34	29.1	16.2
27	Me'phaa	F	7	1.07	16.6	14.49

50	Me’phaa	M	7	1.11	22.3	18.09
6	Me’phaa	F	8	1.02	18	17.3
49	Me’phaa	M	9	1.15	24.7	18.67
28	Me’phaa	M	6	0.97	15.1	16.04
24	Me’phaa	F	6	1.03	19	17.9
4	Me’phaa	M	5	0.86	17.3	23.39 *
51	Me’phaa	M	5	0.99	17.8	18.16
13	Me’phaa	F	8	1.17	21.5	15.7
63	Me’phaa	F	6	1.09	15.6	13.13
45	Me’phaa	F	10	1.24	25.2	16.38
22	Me’phaa	F	10	1.31	44	25.63 *
23	Me’phaa	F	8	1.05	18.3	16.59
10	Me’phaa	F	7	1.07	21	18.34

Supplementary Table S2. Table containing anthropometric data, including height, weight, and BMI from children of both groups [79]. * corresponds to BMI of children with obesity
